# Association of cytomegalovirus infection with hypertension risk: a meta-analysis

**DOI:** 10.1007/s00508-016-0977-x

**Published:** 2016-03-15

**Authors:** Jing Hui, Yuan-yuan Qu, Na Tang, Yong-min Liu, Hua Zhong, La-mei Wang, Qian Feng, Zhen Li, Fang He

**Affiliations:** 1 Department of Pathophysiology/Key Laboratory of Education Ministry of Xinjiang Endemic and Ethnic Diseases, Medical College of Shihezi University, Shihezi, China; 2 Department of Emergency and Critical Care Medicine, The First Affiliated Hospital of Medical College of Shihezi University, Shihezi, China; 3 Centre of Medical Functional Experiments, Medical College of Shihezi University, Shihezi, China

**Keywords:** Cytomegalovirus, Essential hypertension, Meta-analysis

## Abstract

**Background:**

Information regarding association between cytomegalovirus (CMV) infection and essential hypertension (EH) risk is not consistent across studies. Therefore, we conducted a meta-analysis to investigate the association in detail.

**Methods:**

We comprehensively searched the published literature from the PubMed and Embase databases for any study analyzing the association between CMV and EH risk. A random-effects model was used to calculate the pooled odds ratio (OR) with 95 % confidence interval (CI).

**Results:**

Three studies involving 9657 patients were included in the meta-analysis, and the results showed a significantly increased risk of EH in patients with CMV infection. Overall, 79.3 % of the hypertension patients were CMV-positive, which was significantly higher than the percentage for controls (OR = 1.39, 95 % CI = 0.95–2.05, *P* = 0.017). There was significant heterogeneity among the studies included (I^2^ = 70.5 %). The funnel plot and Egger’s test also indicated no publication bias.

**Conclusions:**

The results showed a significant association between CMV and EH, which indicates that CMV infection is a possible cause of EH.

## Background

Essential hypertension (EH) is the most common form of hypertension [1] and is a major risk factor for cardiovascular, cerebrovascular, and renal diseases. Hypertension is a multifactorial disease [2], and its development involves both genetic and environmental factors; however, the specific mechanism and risk factors remain unclear.

As a member of the human *Herpesviridae* family, cytomegalovirus (CMV) contains double-stranded DNA [3] and establishes a latent infection that can persist for the lifetime of the host. Despite being nearly ubiquitous in the population [4], overt human CMV disease in adults is typically restricted to immunocompromised individuals [5–7]. In healthy individuals, both primary infection and the reactivation of latent virus rarely cause any significant clinical symptoms owing to the robust immune response of the host.

CMV infection is associated with various chronic inflammatory diseases, including cardiovascular diseases (CVDs) [8] such as myocarditis, atherosclerosis, and coronary artery disease [9–11]. Hypertension is an important cause of CVDs, and recent studies [12–18] have shown that patients with a CMV infection have an increased risk of EH; however, the association between CMV and EH remains unclear, and thus the effect of CMV infection on blood pressure is controversial. Therefore, we performed the present meta-analysis to more comprehensively investigate the association between CMV and EH.

## Materials and methods

### Literature search and selection

We comprehensively searched the PubMed and Embase databases up to July 2015. The search key words used included “EH or hypertension”, “blood pressure”, and “CMV”. Relevant articles in the reference lists of the published literature were also searched manually for other potential studies.

### Inclusion and exclusion criteria

Studies were included in the meta-analysis if they met the following criteria (1) case-control study, (2) investigated the positive rate of CMV in hypertension patients and controls, (3) hypertension is defined as systolic blood pressure (SBP) or diastolic blood pressure (DBP) of 140 or 90 mmHg, respectively, (4) not an animal study. Studies were excluded if insufficient details were reported to be able to perform the meta-analysis.

### Data extraction

Two authors (JH and Y-YQ) extracted the data independently. In cases where any data were lacking from an article, the authors of selected studies were contacted directly for the missing data. The two reviewers came to an agreement before the final analysis. The following information was extracted from each study: first author’s name, year of publication, country, ethnicity, CMV detection method, patient characteristics, the sample sizes of cases and controls, and the relationship between CMV and EH.

### Statistical analysis

We performed statistical analyses using Stata statistical software ver.12.0. (Stata Corporation, College Station, TX, USA). Meta-analysis was conducted by combining odds ratios (ORs) with corresponding 95 % confidence intervals (CIs) for the association between the CMV-positive rate of hypertension patients and controls. Heterogeneity across all selected studies was evaluated using the Q-test and the I^2^ statistic (range 0–100 %) [19] and was judged to be significant when *P* < 0.1 or I^2^ > 50 %, respectively. We select the random-effects model in the analysis if significant heterogeneity was observed across studies; otherwise, the fixed-effects model was used. Sensitivity analysis was performed with the random-effects model to evaluate the stability of the crude results by removing one study at a time. The Begg’s funnel plot and Egger’s linear regression test were performed to determine the level of potential publication bias [20, 21]. A *P*-value lower than 0.05 was considered statistically significant.

## Results

### Characteristics of studies included

**Fig. 1:**
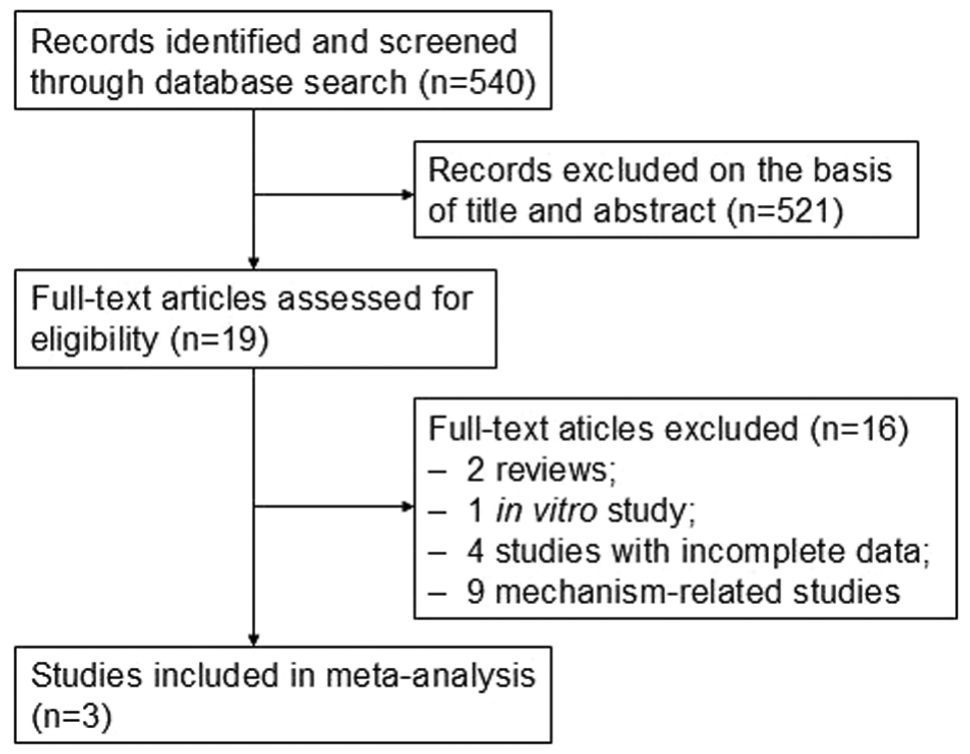
Flow diagram of the study selection process

A total of three articles [12, 13, 16] were included in the meta-analysis, comprising 2347 cases and 7310 controls. All of the studies used enzyme-linked immunosorbent assay (ELISA), and one study [12] used a combination of ELISA and polymerase chain reaction (PCR). ELISA was used to determine the titer of anti-HCMV IgG antibodies, and quantitative PCR was used to test the HCMV DNA copy number. The flow diagram for the search process is shown in Fig. 1. The characteristics of the selected studies are given in Table 1.

**Table 1:** Details of studies included in the meta-analysis

Reference	Year	Ethnicity/Country	CMV detection	*N*	Male (%)	Cases (*n* = 2347)		Controls (*n* = 7310)		Result
						CMV+	CMV-	CMV+	CMV-	
[12]	2014	Han/China	ELISA, PCR	800	42.8	433	49	288	30	CMV antibody titers associated with blood pressure and hypertension
		Kazakh/China		800	42.0	452	15	311	22	CMV seropositivity associated with hypertension in males
[16]	2013	Iran	ELISA	1754	49.2	442	17	1193	102	Pathogen burden not associated with hypertension
[13]	2012	USA	ELISA	6303	47.3	534	405	2902	2462	CMV seropositivity associated with hypertension in women

*ELSA* enzyme-linked immunosorbent assay, *PCR* polymerase chain reaction, *CMV* cytomegalovirus

### Meta-analysis results

**Fig. 2:**
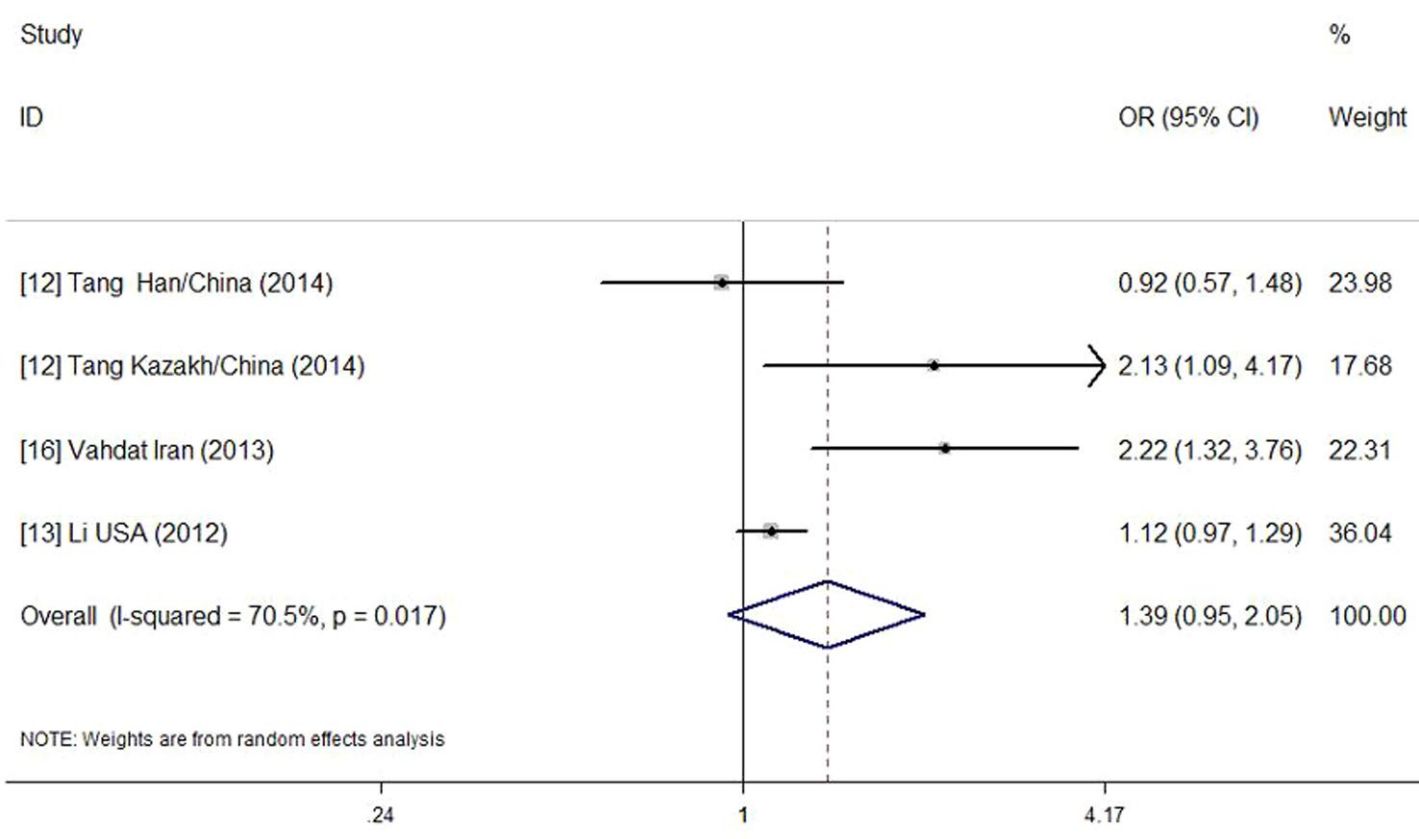
Forest plot of the cytomegalovirus (CMV)-positive rate in hypertension patients versus controls. *OR* odds ratio, *CI* confidence interval

We pooled data from all studies included and analyzed the association between CMV and EH risk. A total of 79.3 % of the hypertension patients were CMV-positive compared with 64.2 % of the controls. The pooled OR was 1.39 (95 % CI = 0.95–2.05, *P* = 0.017). We used a random-effects model, given the significant heterogeneity (I^2^ = 70.5 %) observed across studies. The overall result of the meta-analysis is shown as a forest plot in Fig. 2.

### Sensitivity analysis

**Fig. 3:**
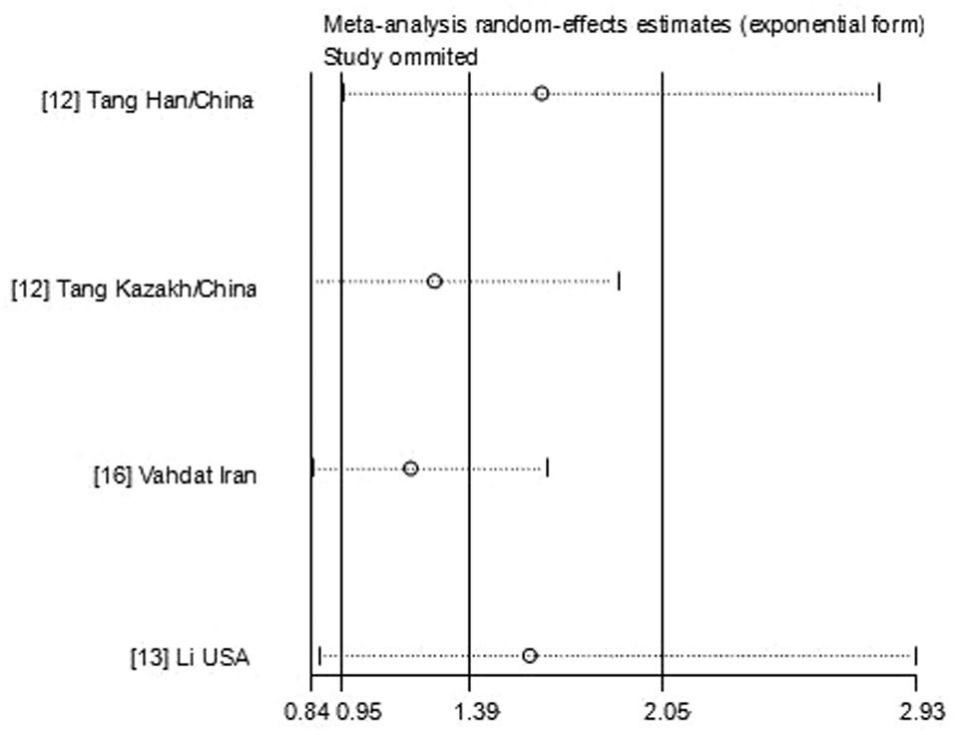
Sensitivity analysis

Sensitivity analysis was conducted to evaluate the stability of the crude results. The results showed that no single study substantially affected the stability of the crude results, given the lack of change in the ORs after exclusion of one study at a time (Fig. 3). Therefore, the results of this meta-analysis were deemed to be reliable.

### Publication bias

**Fig. 4:**
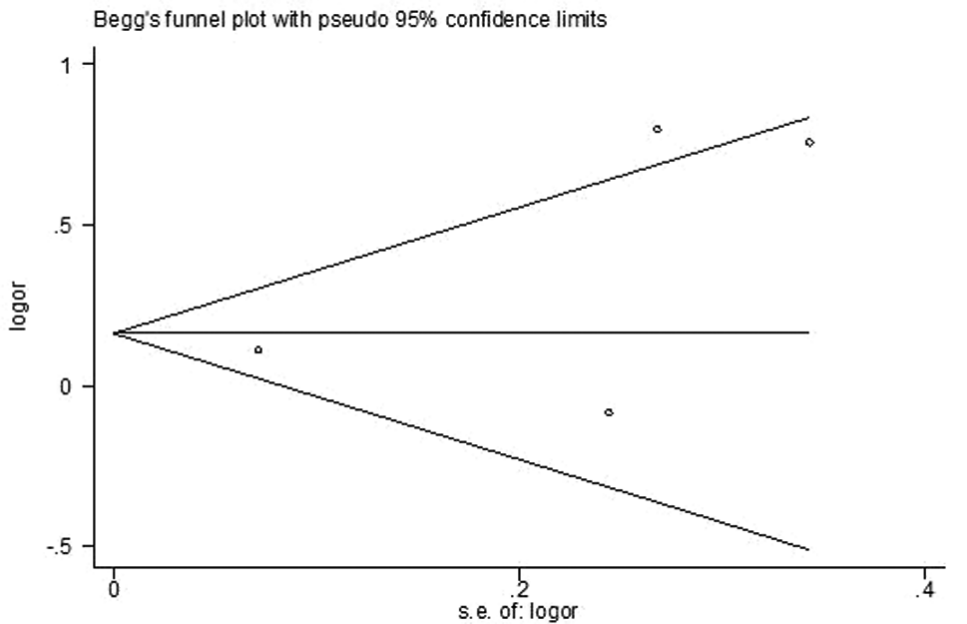
Funnel plot to evaluate potential publication bias in studies evaluating the association between cytomegalovirus (CMV) and essential hypertension risk

Begg’s funnel plot and Egger’s test were used to evaluate the potential publication bias. Although Begg’s funnel plot was not symmetrical, the Egger’s linear regression test indicated that there was no significant publication bias (Fig. 4; *P* = 0.361).

## Discussion

CMV infection has been reported to be associated with hypertension [12–18]. Regardless of the approach used, both negative and positive associations have been reported; therefore, the role of CMV in hypertension remains controversial. Discrepancies between different studies might be due to differences in sample size, age, ethnicity, region, dietary habits, climate, lifestyles, and immune responses to CMV infection, among other factors. Besides, the seroprevalence is considerably high in China [12] to other evaluated studies’ countries, we believe that low socioeconomic status is also a factor affecting the seropositivity [22] in most Asian countries that are still developing countries, especially China.

When a human is first infected, CMV replicates in many different cells. The virus can remain a latent state for the life of its host. In most immunocompetent individuals, the CMV infection is mild or even asymptomatic. Viruses modulate the intracellular environment so that it is optimized to support the viral life cycle. With heightened stress or immunosuppression, however, latent CMV can be reactivated reinitiating productive replication and causing clinical problems, especially in immunosuppressed or immunocompromised patients. Moreover, CMV infection has been shown to be associated with the increased arterial blood pressure via stimulation with renin and cytokine production in mice [23].

Recently, several previous studies have investigated the role of CMV seropositivity as a risk factor for hypertension. In a Chinese cohort, Tang et al. [12]. showed that CMV seropositivity was significantly associated with hypertension after adjustment for age in Kazakh males but not in females. However, in another study cohort [13], CMV seropositivity was associated with hypertension in females before, but not after, adjusting for age. By contrast, another study in a Chinese cohort [14] showed that plasma CMV deoxyribonucleic acid (DNA) copy number was associated with hypertension.

Few studies have examined the CMV antibody titer as a risk factor for hypertension. One study [15] showed that CMV antibody titers were independent determinants for SBP and DBP elevation in young males but not in females. By contrast, Tang et al. [12] demonstrated that, in both Han Chinese males and females, CMV antibody titers were significantly independently associated with hypertension. However, Vahdat et al. [16] failed to show any significant association between viral infections and hypertension in patients from Iran.

In this meta-analysis, we selected three studies [12, 13, 16], including 2347 cases and 7310 controls; all the studies included were population-based cross-sectional studies. We constructed a forest plot to obtain the pooled OR and demonstrated that patients with CMV infection showed a higher risk of EH. Although the mechanism underlying how CMV might cause EH remains unclear, there are several possible explanations for this link. First, oxidative stress and inflammation might play a role [24, 25]. Reactive oxygen species (ROS) have been shown to be generated in response to CMV infection [26] and are also directly involved in vasoconstriction [27–30] and vascular inflammation [31]. CMV infection can induce an inflammatory reaction through modulation of inflammatory mediators that evoke vasoconstriction, such as cytokines, chemokines, and adhesion molecules [32–34]. Second, CMV might influence hypertension via regulation of the renin-angiotensin system. CMV infection has been shown to induce the generation of angiotensin II (AngII) [25], which reacts with endothelial nitric oxide synthase to promote vasoconstriction; this may also enhance the production of ROS [35, 36]. An in vivo experimental study showed that CMV infection increased arterial pressure and further stimulated the expression of renin and increased AngII levels in the blood and arterial tissues [23], which can lead to arterial constriction. Third, the immune response triggered by CMV infection could itself lead to hypertension. Indeed, suppression of the immune system has been shown to depress blood pressure in both experimental animals and humans [37]. Finally, genomic-related changes could be involved in the mechanism. In particular, DNA methylation of gene promoter region [38–40] and the corresponding changes in mRNA expression [14] are also involved in regulating blood pressure. The studies included there are not enough, so we need more prospective studies to prove the link.

The results of the present meta-analysis suggest that CMV may be a contributing factor to the development of EH. However, there are limitations to the meta-analysis that should be acknowledged. First, although the pooled sample size of the studies included was sufficiently large to carry out the meta-analysis, the number of studies included is not as large as we had hoped for in order to perform a comprehensive analysis. In particular, this limited our ability to conduct a subgroup analysis to explore the influence of sample size, ethnicity, age, or other factors and to explore the nature of the observed heterogeneity. Therefore, these findings should be interpreted with caution, and more studies are needed to confirm the results of this meta-analysis.

## Conclusions

The results showed a significant association between CMV and EH, which indicates that CMV infection is a possible cause of EH.

### Source of support

This project was supported by the Joint Funds of the National Natural Science Foundation of China (No.U1403123) and the Ministry of Major Science & Technology of Shihezi University (Nos.gxjs2013-zdgg04, gxjs2013-zdgg04-1, gxjs2013-zdgg04-2, gxjs2013-zdgg04-3, and gxjs2013-zdgg04-4) and the Xinjiang Graduate Student Research Innovation Project (No. XJGRI2015054). No conflict of interest exists in this study.
